# Effects of virtual reality-based motor control training on inflammation, oxidative stress, neuroplasticity and upper limb motor function in patients with chronic stroke: a randomized controlled trial

**DOI:** 10.1186/s12883-021-02547-4

**Published:** 2022-01-11

**Authors:** Chien-Yu Huang, Wei-Chi Chiang, Ya-Chin Yeh, Shih-Chen Fan, Wan-Hsien Yang, Ho-Chang Kuo, Ping-Chia Li

**Affiliations:** 1 Department of Occupational Therapy, I-Shou University, Yanchao Dist., Kaohsiung, 824 Taiwan, R.O.C.; 2 School of Occupational Therapy, National Taiwan University, Zhongzheng Dist., Taipei, 100 Taiwan, R.O.C.; 3 Department of Occupational Therapy, Shu-Zen Junior College of Medicine and Management, Kaohsiung, 741 Taiwan, R.O.C.; 4 Institute of Allied Health Sciences, College of Medicine, National Cheng Kung University, Tainan, 701 Taiwan, R.O.C.; 5 Tan-Chi International Technology Co., Ltd, 824 Kaohsiung, Taiwan, R.O.C.; 6 Kawasaki Disease Center and Department of Pediatrics, Kaohsiung Chang Gung Memorial Hospital and Chang Gung University College of Medicine, Kaohsiung, 824 Taiwan R.O.C.

**Keywords:** Virtual reality (VR), Stroke rehabilitation, Inflammation, Oxidative stress, Neuroplasticity, Heme oxygenase-1 (HO-1), 8-hydroxydeoxyguanosine (8-OHdG), Brain-derived neurotrophic factor (BDNF)

## Abstract

**Background:**

Immersive virtual reality (VR)-based motor control training (VRT) is an innovative approach to improve motor function in patients with stroke. Currently, outcome measures for immersive VRT mainly focus on motor function. However, serum biomarkers help detect precise and subtle physiological changes. Therefore, this study aimed to identify the effects of immersive VRT on inflammation, oxidative stress, neuroplasticity and upper limb motor function in stroke patients.

**Methods:**

Thirty patients with chronic stroke were randomized to the VRT or conventional occupational therapy (COT) groups. Serum biomarkers including interleukin 6 (IL-6), intracellular adhesion molecule 1 (ICAM-1), heme oxygenase 1 (HO-1), 8-hydroxy-2-deoxyguanosine (8-OHdG), and brain-derived neurotrophic factor (BDNF) were assessed to reflect inflammation, oxidative stress and neuroplasticity. Clinical assessments including active range of motion of the upper limb and the Fugl-Meyer Assessment for upper extremity (FMA-UE) were also used. Two-way mixed analyses of variance (ANOVAs) were used to examine the effects of the intervention (VRT and COT) and time on serum biomarkers and upper limb motor function.

**Results:**

We found significant time effects in serum IL-6 (*p* = 0.010), HO-1 (*p* = 0.002), 8-OHdG (*p* = 0.045), and all items/subscales of the clinical assessments (*p*s < 0.05), except FMA-UE-Coordination/Speed (*p* = 0.055). However, significant group effects existed only in items of the AROM-Elbow Extension (*p* = 0.007) and AROM-Forearm Pronation (*p* = 0.048). Moreover, significant interactions between time and group existed in item/subscales of FMA-UE-Shoulder/Elbow/Forearm (*p* = 0.004), FMA-UE-Total score (*p* = 0.008), and AROM-Shoulder Flexion (*p* = 0.001).

**Conclusion:**

This was the first study to combine the effectiveness of immersive VRT using serum biomarkers as outcome measures. Our study demonstrated promising results that support the further application of commercial and immersive VR technologies in patients with chronic stroke.

**Supplementary Information:**

The online version contains supplementary material available at 10.1186/s12883-021-02547-4.

## Background

Stroke affects 80 million people annually and claims 5.5 million lives and 116 million disability-adjusted life years worldwide. This number is gradually increasing, making stroke a serious global healthcare issue [1]. Approximately 85% of stroke survivors exhibit various degrees of motor paralysis [2], and 55 to 75% of patients experience paralysis of an arm [3]. Upper limb paralysis may affect a person participating in activities of daily living [4], increases the burden on the caregiver, and puts an economic pressure on both the patient’s family, and the society.

Rehabilitation is critical and a long-term journey for a patient with stroke. Through motor control and functional training, rehabilitation facilitates post-stroke motor recovery and improves patient participation in daily life. Implementing motor control training for a patient to relearn motor skills also facilitates neuroplasticity. Neuroplasticity is defined as the brain capacity of undergoing functional and structural changes through growth and reorganization [5]. Moreover, neuroplasticity helps motor recovery. Previous studies revealed that enhancing adaptive neuroplasticity and promoting brain activity help improve motor function [6, 7]. Therefore, motor control training plays a critical role in rehabilitation in patients with stroke.

However, a challenge of motor control training is the low motivation and compliance with the intervention, which largely affects its effectiveness. To improve the patients’ rehabilitation motivation, rehabilitation activities involving the use of virtual reality (VR) have been developed. VR provides a novel virtual environment and multisensory stimulation that adds an entertainment value to the intervention, and thus may improve an individual’s sense of self-reflection and self-efficacy through the processes of adaptation and engagement [8].

An immersive VR system, using external devices such as a head-mounted display (HMD) to create a full-body sense of presence in a virtual environment, has been increasingly used in VR-based rehabilitation. Evidence exists for the effectiveness of using an immersive VR system in motor control training. Song and Lee have developed VR activities with immersive VR systems to provide bilateral arm training in patients with stroke [9]. They found that both the immersive VR-based bilateral arm training group and the normal bilateral arm training group showed significant differences in upper limb function between pre- and post-test, without significant differences between groups [9]. Ögün et al. conducted a randomized control trial to examine the effectiveness of Leap Motion-based 3D immersive VR system on upper limb function in patients with ischemic stroke [10]. In both groups (VR and control group), participants showed improved upper limb function overtime. Moreover, the VR group had significantly higher scores in the assessments of Fugl-Meyer Assessment for upper extremity (FMA-UE), Action Research Arm Test (ARAT), the Functional Independence Measure, and the Performance Assessment of Self-Care Skills. Accordingly, immersive VR rehabilitation appeared to be effective in improving upper extremity function and self-care skills.

Moreover, the immersive VR system can be customized or commercialized. Compared to a customized VR system, a commercial VR system requires less developmental costs and fewer guidelines for operation, which may decrease the financial burden and workload for practitioners in clinical practice. Erhardsson et al. have conducted a single case study exploring the potential of using commercial immersive VR system for chronic stroke rehabilitation [11]. Using motor function-related assessments (e.g., ARAT, Box and Block Test, and kinematic measures) as outcome measures, they showed that among six participants, four showed an improvement in ARAT (Tau-U scores = 0.50–0.92). Two participants also showed improvements in kinematic measures. No serious adverse effects of VR-based motor control training (VRT) were observed. Therefore, using a commercialized VR system originally designed for entertainment could be an alternative strategy for VR rehabilitation.

Although the immersive VRT has been shown to improve motor function in patients with stroke, the outcome measures used for VRT were mainly based on motor function measurements (e.g., the ARAT, Box and Block Test, and the FMA-UE) [10–16] or the examination of brain cortical activation (e.g., magnetic resonance imaging [MRI], motor-evoked potentials, computed tomography [CT], and electroencephalography) [17–22]. Few studies have used serum biomarkers as outcome measures. However, several stroke mechanisms related to inflammation, oxidative stress, and neuroplasticity may reflect the neuroprotective effects of motor control training on a molecular level. Such molecular changes could be detected by serum biomarkers. For example, interleukin 6 (IL-6) [23–26] and intercellular adhesion molecule (ICAM-1) could represent the inflammation level [23, 27, 28], 8-hydroxy-2′-deoxyguanosine (8-OHdG) [29, 30] and heme oxygenase-1 (HO-1) could provide the information of oxidative stress [31–35], and brain-derived neurotrophic factor (BDNF) could be an indicator of neuroplasticity [36, 37].

In particular, BDNF is a key factor in neuroplasticity and is involved in brain cell regeneration, reestablishment, and rearrangement of neural connections, and further influences the functional outcome after stroke [37]. A secreted protein extensively expressed in the brain, it can traverse the blood–brain barrier in both directions. Because of its neurotrophic effects and detectable changes in serum, restoration or increase of BDNF levels may have a beneficial effect in neurologic disorders. However, the relationship between the serum BDNF concentration and short-term functional outcomes is controversial. Three studies have indicated that low serum BDNF at admission was significantly associated with poor functional outcomes at 3 months [38–40], and two studies have shown non-significant results [41, 42], although one of the studies also indicated that the low BDNF concentration was associated with poor functional outcomes at 2 and 7 years [42]. Since tissue repair may be followed by inflammation resolution and homeostasis restoration, which is a long-lasting process, the focus should lie on the changes of BDNF expression at the chronic stage rather than the acute stage.

Moreover, investigating changes in serum molecules along with conventional assessments may increase the understanding of the pathological mechanisms underlying stroke at the chronic stages. In addition, in comparison to CT and MRI, serum markers are easier to collect, detect, and analyze. Therefore, we suggest investigating the changes of serum biomarkers to reveal the effects of post-stroke motor control training at a molecular level, and reflect the changes on inflammation, oxidative stress, and neuroplasticity.

In summary, our study aimed to use a commercial HMD immersive VR system combined with therapist-designed tasks to investigate the effects of VRT on serum markers of inflammation, oxidative stress and neuroplasticity, and upper limb motor function in patients with chronic stroke.

## Methods

### Participants

**Fig. 1:**
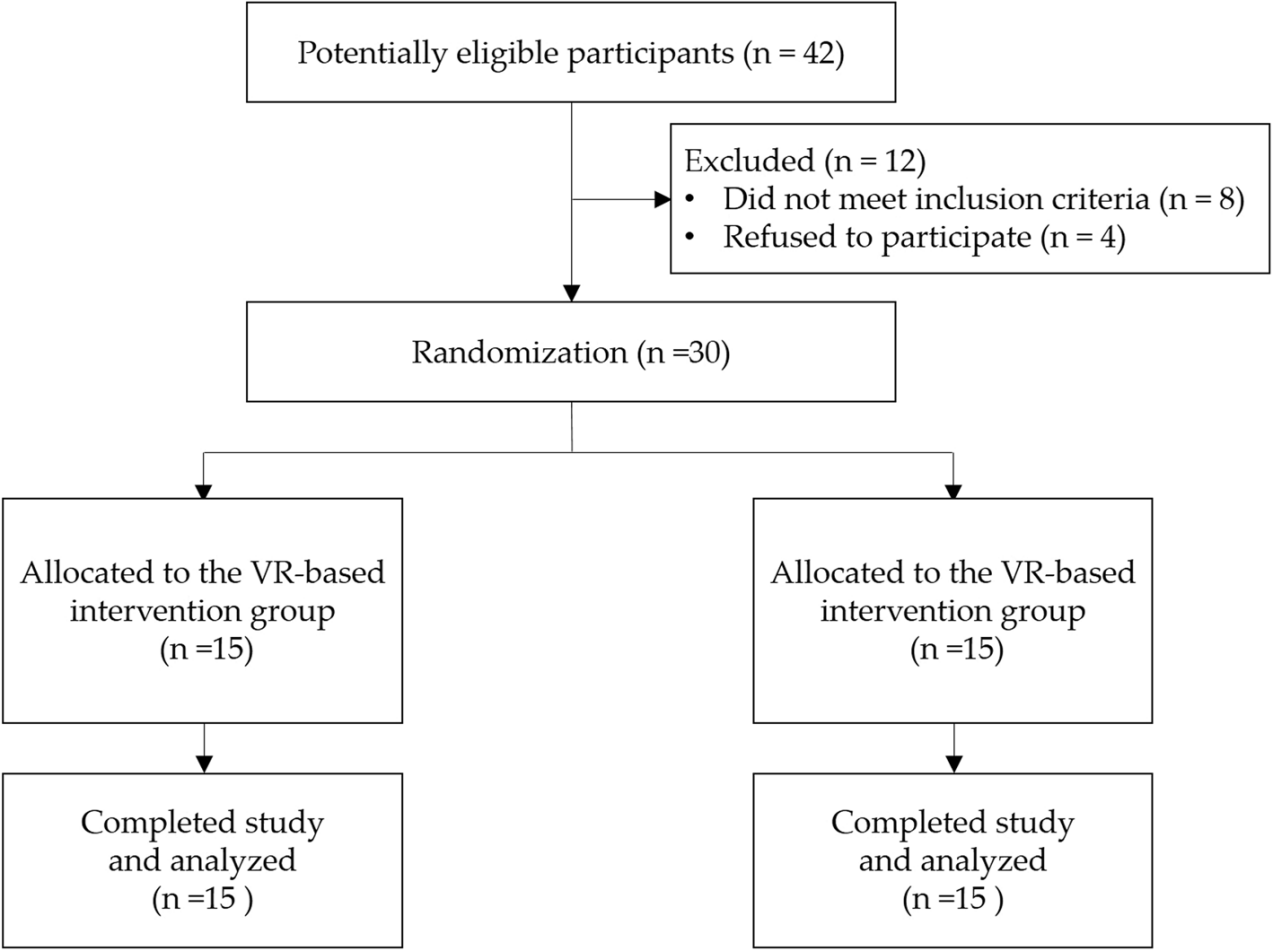
Flow diagram of the participant inclusion

Individuals with stroke were recruited from a hospital in southern Taiwan. The inclusion criteria were (1) age between 20 to 75 years old, (2) stroke onset > 3 months, (3) Brunnstrom stage > 3, (4) a diagnosis of stroke confirmed with computerized tomography (CT) or magnetic resonance imaging (MRI)scans, (5) Mini-Mental State Examination score (MMSE) > 18, indicating being able to understand the instructions and (6) having no other neurological disorders as comorbidities. The exclusion criteria were as follows: (1) participated in other rehabilitation-related or clinical trials within 3 months of the experiment, (2) sensory apraxia, (3) severe impairments in vision or visual perception such as hemi-neglect, (4) receiving warfarin or vitamin K antagonist treatment, (5) high risk of epilepsy, (6) failure to cooperate with the researcher to execute VR activities, and (7) refusal to undergo the blood test. Forty-two potentially eligible participants were invited by the therapist, and 30 were eligible to participate in the study (Fig. 1). This study was approved by the Institutional Review Board of E-Da Hospital with Clinical Trials Approval Certificate Number EMRP-108-006 (07/04/2019) and was registered in the WHO International Clinical Trials with Portal Number ChiCTR2100047853 (27/06/2021). Informed consent was obtained from all participants involved in the study. All study procedures were conducted in accordance with the Declaration of Helsinki.

### Design

This study was an assessor-blinded, randomized controlled trial with pre-intervention and post-intervention assessments. Full details of the trial protocol can be found in the Additional file 3. Participants were randomly equally allocated to the conventional occupational therapy (COT) and virtual reality training (VRT). Randomization procedures were performed using a randomization table generated online (freely available at http://www.randomizer.org/). The allocation sequence was attained by a researcher not involved in the intervention and evaluation. Clinical assessments were performed within one week before and after interventions by another therapist not involved in training and blinded to the purpose and group allocation. Questionnaires evaluating user’s experience were assessed after each session. Blood collection was also conducted before and after interventions by licensed medical personnel at the same hospital.

### Intervention

All participants received 16 sessions of intervention for 60 min/day, 2 to 3 days/week, as well as attended regular occupational therapy. Each session was supervised by an occupational therapist. In the COT group, conventional instruments such as the peg board, climbing ladder, and stacking cones were used for upper limb training. In the VRT group, participants executed VR-based activities conducted by the same therapist. Six to ten tasks were assigned in each session. The commercial immersive VR headset developed by HTC VIVE (HTC Corporation, New Taipei City, Taiwan) was used in this study. The VR equipment contains an HMD device, two controllers and two infrared laser emitter units. The VR equipment was installed in a room without external disturbances, and the virtual environment was set in a 6 m^2^ physical space. At the beginning of each session, the participant would sit in the center of the set zone and was assisted to wear the HMD. After the participant confirmed that the sight and sound were clear and comfortable, the controllers were handed to the participant.

Twenty VR scenes (see Additional file 1) from commercial games were selected for the participants. Only the last VR scene was purchased, and the rest were free or partly free. Participants able to walk experienced all the tasks, while those unable to walk experienced 15 scenes (exclusion of Scenes 6, 8, 10, 18, and 19). Scenes 6, 8, 10, 18, and 19 required either standing in a fixed position, or moving a few steps to complete the tasks. Selected scenes were chosen based on the original upper limb activities. Participants could accomplish the first-contact task with the help of their unaffected hand, and then they were encouraged to use the affected hand or both hands at same time. Tasks in several scenes (Scenes 10, 12, and 17) could be completed using only one hand, while tasks in other scenes (Scenes 3, 8, 18, and 19) required bilateral coordination of both hands. Upper limb movements in most scenes involved aiming, shooting, hitting, waving arms, punching, and throwing objects. Upper limb movements in Scenes 18 and 19 were more complex, since tasks in the two scenes simulated daily life activities, such as shopping, pouring water, and blending drinks.

Participants’ activity performance and information were recorded, including training intensity, duration, game scores, level completed, and invalid activities. This information allowed the therapist to assign the tasks, adjust the difficulty in the VR game settings, and even design a new way to play the game for each participant according to their needs and capacities.

### Outcome measures

#### Clinical assessments

The Fugl-Meyer Assessment for upper extremity (FMA-UE) and active range of motion (AROM) were assessed to exam motor function changes in patients with stroke. The FMA-UE is widely used to examine motor impairment of upper limb performance of motor, sensation, coordination, range of motion, and speed. It is a highly reliable and valid assessment tool. The score of the FMA-UE ranges from 0 to 66, with a higher score indicating better function. A total score and four subscale scores of the FMA-UE were used in this study. The subscale scores included upper extremity (18 items; 0 to 36), wrist (5 items; 0 to 10), hand (7 items; 0 to 14), and coordination/speed (3 items; 0 to 6). AROM was used to evaluate isolated joint motion by the participant self reporting reaching maximal capacity without pain. Five AROM items including shoulder flexion, elbow extension, wrist extension, forearm supination and pronation were assessed with a goniometer.

#### Serum sampling for molecular biomarkers

Blood samples from all participants were collected before and after 16 weeks of intervention. Since BDNF protein expression may be affected by age, circadian rhythm, and menstrual cycle [43], blood was drawn at the same time point before and after intervention. The intervention lasted approximately one month, which met the menstrual cycle of the only woman who has not yet undergone menopause. Whole blood was maintained at 40 °C for 30 min and then centrifuged at 1000 x g for 15 min, with the supernatant transferred to several microfuge tubes to avoid repeated freeze–thaw cycles. Serum samples were stored at − 80 °C and each aliquot was used only once. Serum IL-6 (Invitrogen Corp., Carlsbad, CA, USA), ICAM-1 (Invitrogen Corp., Carlsbad, CA, USA), HO-1 (Aviva Systems Biology, San Diego, CA, USA), 8-OHdG (Aviva Systems Biology, San Diego, CA, USA), and BDNF (Invitrogen Corp., Carlsbad, CA, USA) were measured by the respective commercial enzyme linked immunoassay (ELISA) kit following the manufacturer’s standard protocols. All samples were analyzed in triplicate.

#### Satisfaction and safety

Side effects, simulation motion sickness symptoms, and enjoyment were recorded for each session. For each session, participants in the VRT group had to complete the user’s experience questionnaire, including Simulator Sickness Questionnaire (SSQ), Borg Scale of Perceived Exertion (RPE) scale, and a few simple rating questions about physical and mental health conditions, satisfaction, and favorite game. After the intervention, participants had to fill out 18 questions related to satisfaction of the VR training.

The SSQ includes 18 possible side effects of immersive VR activities. Each item is rated on a 4-point scale: no discomfort, slight discomfort, moderate discomfort, and severe discomfort. The total score ranges from 0 to 54 [44].

The RPE scale is designed by the Swedish psychologist Gunnar Borg to estimate the intensity of exercise based on the user’s own feelings during the activity. The score ranges from 6 to 20, representing the feeling of no exertion to maximum effort. Although subjective, previous studies have shown that multiplying the RPE value by 10 is highly correlated with the actual heart rhythm [45].

### Statistics analysis

Sample size was estimated using G*Power software and based on previous research [10]. The effect size 0.79 proposed by Guillermo was used in this study [46], a significance level of 5% and a test power of 80% were applied.

Descriptive analysis was used to describe characteristics of participants and illustrate participants’ perception of participating in VRT. Demographic data between groups were compared using the chi-square test for categorical variables and Mann-Whitney *U* test for continuous variables. Two-way mixed analyses of variance (ANOVAs) were used to examine the effects of the intervention (VRT and COT) and time on the upper limb motor function and serum biomarkers. Further, we used the Wilcoxon signed rank test and the Mann-Whitney *U* test to compare independent-samples (pre- and post-treatment effects in each group) and paired-samples (pre- and post-treatment effects within groups), respectively. Significant models were further assessed by Bonferroni post-hoc tests to compare differences between each pair of the five variables in either clinical assessment (FMA and AROM).

Spearman’s correlations and Kruskal-Wallis test were used to examine the relationships between demographic characteristics (e.g., age, subtype of stroke, education, and cognitive function) and motor performance and serum biomarkers. Correlations between clinical outcomes and biomarker concentration were assessed using Spearman’s correlation coefficient. The alpha level was set at 0.05. Data were analyzed using statistical software SPSS 18.0 (IBM Corp., Chicago, NY, USA).

## Results

### Participant characteristics

Thirty patients with chronic stroke were enrolled in our study. The demographic and clinical characteristics are shown in Table 1; there were no significant differences between the COT and VRT groups (all *p* > 0.05).

**Table 1:** Demographic, neurological, and functional characteristics of the COT and VRT groups at baseline

Variables	COT group (***n*** = 15)	VRT group (n = 15)	***p***
Age (years), mean (SD)	58.33 ± 11.22	50.80 ± 12.32	0.093
Sex (female/male), n (%)	11/4 (73/27)	9/6 (60/40)	0.439
Years of education (years), mean (SD)	10.40 ± 6.092	11.20 ± 3.509	0.863
Body mass index, mean (SD)	24.46 ± 3.15	24.42 ± 4.04	0.971
Time since stroke (months), mean (SD)	17.91 ± 21.25	36.20 ± 42.38	0.071
Affected extremity, n (%)			
Right/Left	5/10 (34/66)	6/9 (40/60)	0.705
Dominant/Non-dominant	5/10 (34/66)	8/7 (53/47)	0.269
Subtype of stroke, n (%)			
Infarction (TOAST 1/2/3/4/5)/	7/0/3/2/0/3	4/2/2/2/0/5	0.475
Hemorrhage	(46/0/20/13/0/20)	(26/13/13/13/0/33)	
Mini-Mental State Examination	27.93 ± 1.907	26.53 ± 0.354	0.422

*COT* Conventional occupational therapy, *VRT* Virtual reality training; TOAST classification: 1: large-artery atherosclerosis; 2: cardioembolism; 3: small-vessel occlusion; 4: stroke of other determined etiology; 5: stroke of undetermined etiology

The ages of the COT group participants ranged from 28 to 71 years, and the ages of participants in the VRT group ranged from 22 to 70 years. Regarding the stroke subtypes, the major type was ischemic stroke. The maximum and minimum Mini-Mental State Examination scores of both groups were 23 and 30, respectively. The baseline motor function and baseline level of serum molecular biomarkers are listed in Table 2 and Table 3, respectively; no significant differences were observed between groups at the beginning of the trial (all *p* > 0.05).

**Table 2:** Clinical assessment scores in the COT group and VRT group

Upper limb assessment	COT group		VRT group		F	Time	Group
	Pre-test	Post-test	Pre-test	Post-test	Time x Group		
FMA-UE							
Shoulder/Elbow/Forearm	24.53 ± 7.87	24.87 ± 7.75	26.33 ± 4.94	27.53 ± 4.16^β^	8.895^α^	11.831^α^	1.045
Wrist	7.33 ± 8.13	8.00 ± 12.90^β^	8.13 ± 1.80	8.87 ± 1.92	0.028	17.441^αα^	1.036
Hand	8.53 ± 4.14	9.00 ± 4.14	10.20 ± 2.88	11.00 ± 2.54	1.750	9.645^α^	3.541
Coordination/Speed	4.07 ± 2.63	4.07 ± 2.63	4.73 ± 2.05	5.07 ± 2.09	4.375	4.375	1.264
Total	44.47 ± 16.60	45.53 ± 16.39^β^	49.40 ± 9.02	52.47 ± 9.11^β^	9.669^α^	42.422^αα^	1.768
AROM							
Shoulder Flexion	139.00 ± 59.95	141.20 ± 59.53^β^	138.33 ± 24.47	150.93 ± 24.47^ββ^	17.975^αα^	54.218^αα^	0.065
Elbow Extension	7.07 ± 2.94	7.53 ± 3.00	8.60 ± 2.33	10.60 ± 3.50	3.130	10.162^α^	9.840^α^
Wrist Extension	38.73 ± 30.80	39.87 ± 31.00	39.00 ± 18.82	47.40 ± 19.87^β^	3.661	5.287^α^	0.161
Forearm Pronation	68.93 ± 16.23	70.73 ± 16.70	75.07 ± 13.48	78.53 ± 12.81^β^	1.316	22.323^αα^	4.699^α^
Forearm Supination	53.00 ± 30.39	53.47 ± 30.63	64.80 ± 18.96	68.73 ± 13.85	2.756	4.763^α^	3.197

^α^, ^αα^ The effect of group and time examined by mixed model design ANOVA were significant (*p* ≤ 0.05) and highly significant (*p* ≤ 0.001)

^β^, ^ββ^ Comparison between pre-test and post-test examined by Wilcoxon signed rank test were significant (*p* ≤ 0.05) and highly significant (*p* ≤ 0.001). COT: conventional occupational therapy; *VRT* Virtual reality training

**Table 3:** Expression levels of serum biomarkers in the COT group and VRT group

Serum biomarkers	COT group		VRT group		F	Time	Group
	Pre-test	Post-test	Pre-test	Post-test	Time x Group		
Inflammation							
IL-6 (pg/mL)	5.36 ± 2.34	4.33 ± 1.80^β^	5.24 ± 3.10	3.95 ± 2.56	0.079	8.770^α^	0.071
ICAM-1 (ng/mL)	430.81 ± 34.36	475.44 ± 93.99	419.80 ± 106.68	433.32 ± 70.39	1.865	2.500	1.059
Oxidative stress							
HO-1 (pg/mL)	48.01 ± 25.23	57.33 ± 25.04^β^	51.87 ± 21.74	64.83 ± 20.89^β^	0.137	14.388^α^	0.528
8-OHdG (pg/mL)	6.60 ± 2.27	6.48 ± 2.05^β^	7.10 ± 2.30	6.56 ± 2.09^β^	0.788	4.837^α^	0.155
Neuroplasticity							
BDNF (ng/mL)	110.96 ± 16.24	109.09 ± 14.37	116.99 ± 11.17	122.11 ± 10.56^β^	2.343	0.584	3.443

^α^ The effect of group and time examined by mixed model design ANOVA were significant (*p* ≤ 0.05). ^β^ Comparison between pre-test and post-test examined by Wilcoxon signed rank test were significant (*p* ≤ 0.05). *COT* conventional occupational therapy, *VRT* virtual reality training

### Effects of the intervention on upper limb motor function

**Fig. 2:**
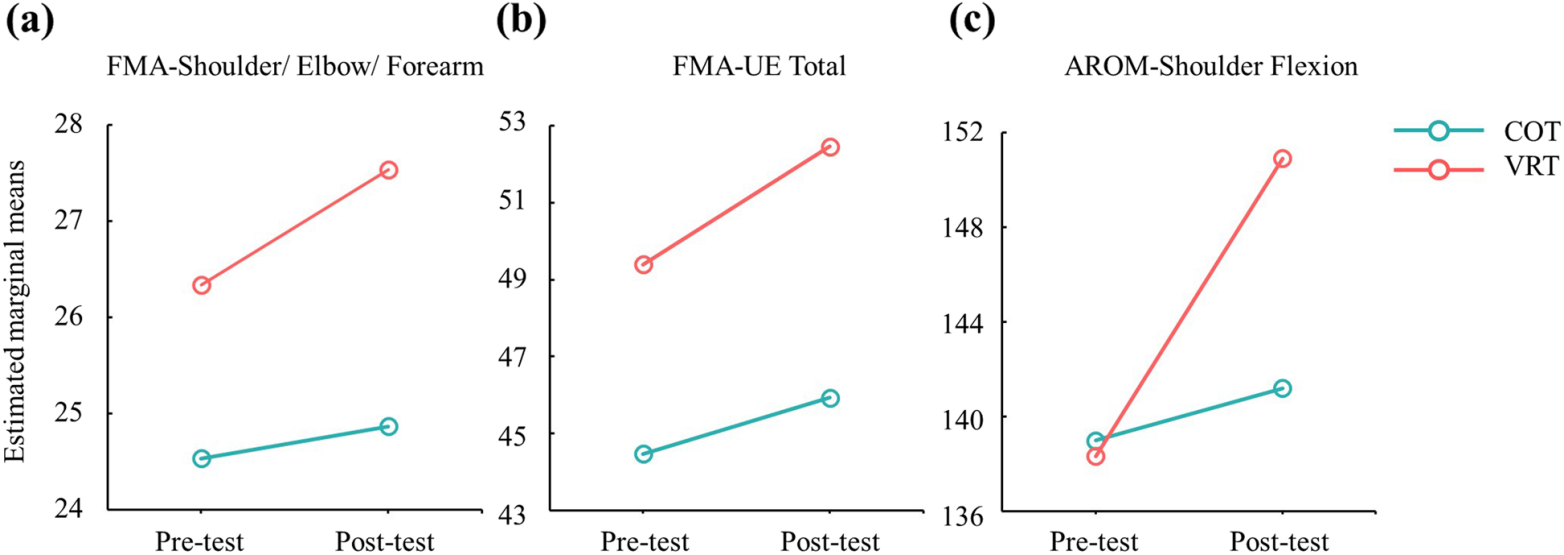
Significant interaction effect between group and time in FMA-UE-Shoulder/Elbow/Forearm (**a**), FMA-UE-Total (**b**), and AROM-Shoulder Flexion (**c**)

The results of motor function are presented in Table 2. Two-way mixed ANOVA revealed a significant time effect for all items of upper limb assessment (F ranges from 4.763 to 54.218, all *p* < 0.05) except FMA-UE-Coordination/Speed (F = 4.375, *p* = 0.055), and a significant group effect for AROM-Elbow Extension (F = 9.840, *p* = 0.007) and AROM-Forearm Pronation (F = 4.699, *p* = 0.048). There was a significant interaction between time and group for FMA-UE-Shoulder/Elbow/Forearm (F = 8.895, *p* = 0.004), FMA-UE-Total score (F = 9.669, *p* = 0.008), and AROM-Shoulder Flexion (F = 17.975, *p* = 0.001). As shown in Fig. 2, the improvement in FMA-UE-Shoulder/Elbow/Forearm, FMA-UE-Total score, and AROM-Shoulder Flexion was significantly higher in the VRT group than in the COT group.

Additionally, we demonstrated the comparison between pre-test and post-test as well as the delta change between groups.

After the intervention, significant improvements were observed in both groups for FMA-UE-Total scores (COT: 3.86%, *p* = 0.021, VRT: 6.60%, *p* = 0.003) and AROM-Shoulder Flexion (COT: 2.87%, *p* = 0.023, VRT: 9.65%, *p* = 0.003). Significant improvement in FMA-UE-Wrist was only observed in the COT group (8.57%, *p* = 0.033), whereas significant improvements in FMA-UE-Shoulder/Elbow/Forearm (6.01%, *p* = 0.035), AROM-Wrist Extension (35.65%, *p* = 0.025), and AROM-Forearm Pronation (4.83%, *p* = 0.038) scores were observed only in the VRT group. Moreover, FMA-UE-Total scores (*p* = 0.046) and AROM-Shoulder Flexion (*p* = 0.001) of the VRT group changed significantly more than those of the COT groups.

**Fig. 3:**
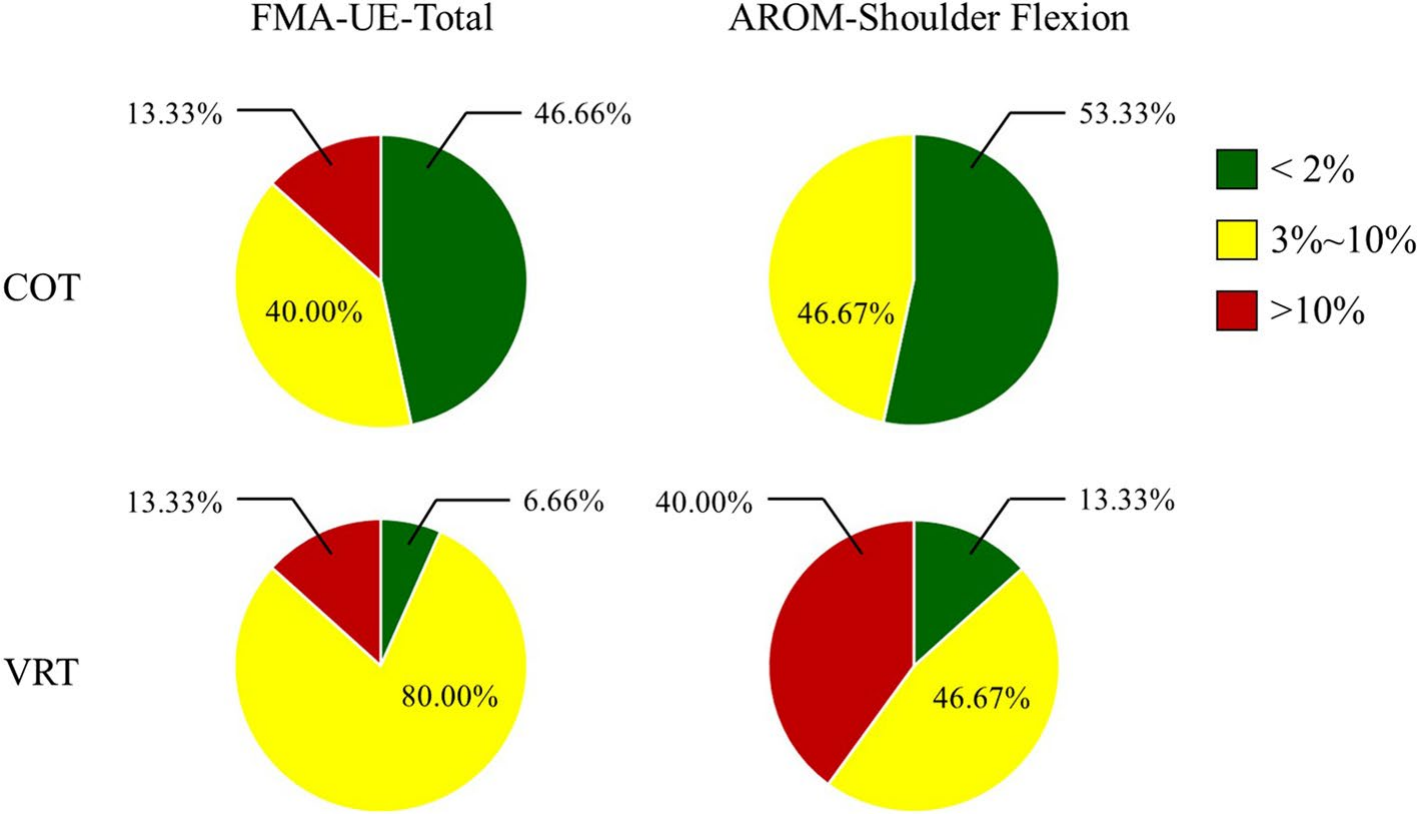
Range of FMA-UE-Total score and AROM-Shoulder Flexion score changes. AROM: active range of motion; FMA-UE: Fugl-Meyer Assessment for upper extremity

Furthermore, we investigated the percentage change of FMA-UE-Total scores and AROM-Shoulder Flexion. A significant difference was found between the COT and VRT group, as shown in Fig. 3. Comparison between groups of FMA-UE-Total (*p* = 0.046) and AROM-Shoulder Flexion showed significant differences (*p* = 0.008). The average FMA-UE-Total score increased by 3.86% in the COT group and 6.60% in VRT group; there was a < 2% increase among most patients in the COT group, whereas 80% of patients in the VRT group showed an increase from 2 to 10%. The average AROM-Shoulder Flexion score increased by 2.87% in COT the group and 9.65% in the VRT group, none of the participants in the COT group exhibited an increase > 10%, whereas 40% of patients in the VRT group showed an increase > 10%.

### Effects of the intervention on serum biomarker levels

Two-way mixed ANOVA revealed a significant time effect for IL-6 (F = 8.770, *p* = 0.010), HO-1 (F = 14.388, *p* = 0.002), and 8-OHdG (F = 4.837, *p* = 0.045). No significant group effects and interactions were found (Table 3).

**Fig. 4:**
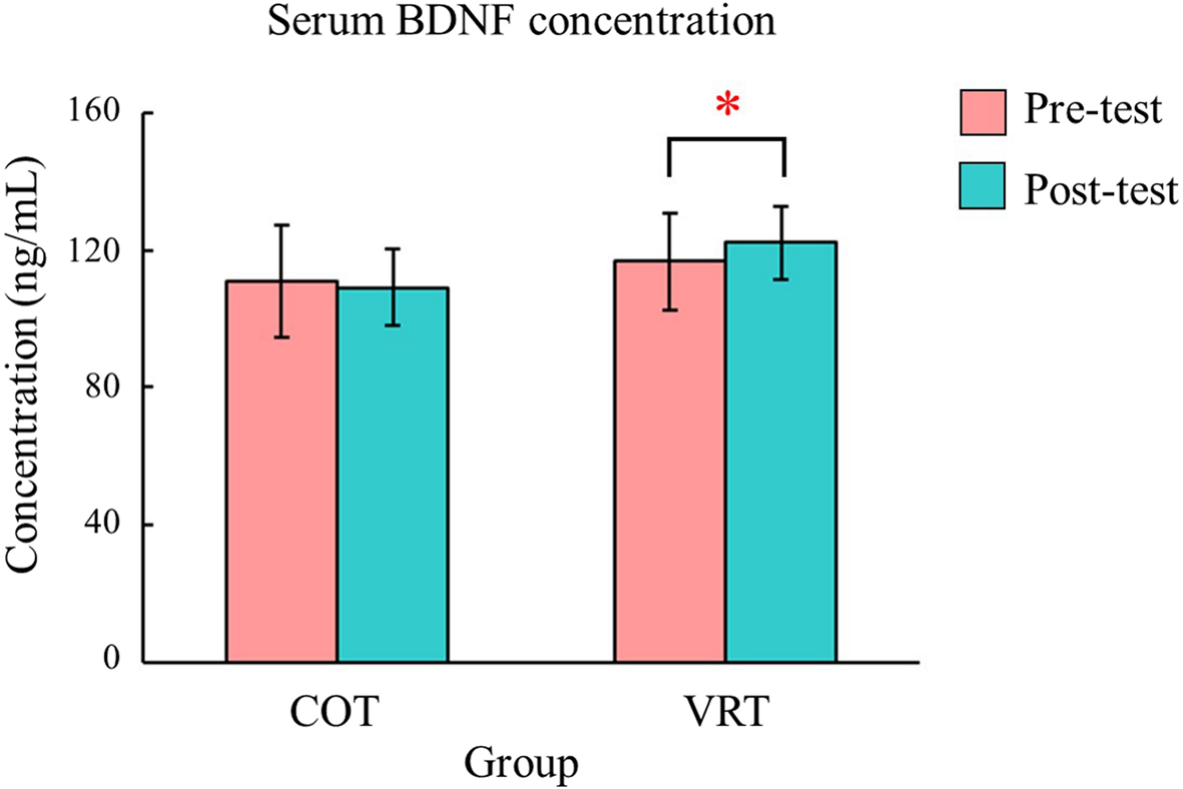
Changes of BDNF levels in the two groups. COT: conventional occupational therapy; VRT: virtual reality training; BDNF: brain-derived neurotrophic factor

Additionally, we demonstrated the comparison between pre-test and post-test as well as the delta change between groups. After intervention, IL-6 levels decreased by 13.57% in the COT group (*p* = 0.016) and 17.96% in the VRT group, but the change in the VRT group was not significant. There were no significant changes in ICAM-1 levels. The concentration of HO-1 tended to increase (COT: 40.99%, *p* = 0.014, VRT: 38.35%, *p* = 0.027) while that of 8-OHdG tended to decrease (COT: 0.51%, *p* = 0.048, VRT: 0.77%, *p* = 0.041) in both the COT and VRT groups. The changes of the abovementioned factors showed a similar trend in both groups, except for BDNF, which decreased slightly in the COT group (0.77%, *p* = 0.103), but increased significantly in the VRT group (4.87%, *p* = 0.023; Table 3 and Fig. 4).

### Relationship between participant characteristics and changes in upper limb motor function and serum biomarker levels

Baseline characteristics including sex, years of education, body mass index, time since stroke, affected side, stroke subtypes, and MMSE score showed no correlation with either the change of serum biomarker levels or the improvements in motor performance (all *p* > 0.05). However, the initial age was negatively correlated with the improvement in FMA-UE-Hand scores (*r* = − 0.407, *p* ≤ 0.05).

### Correlation between upper limb motor function improvement and changes in serum biomarker levels

**Fig. 5:**
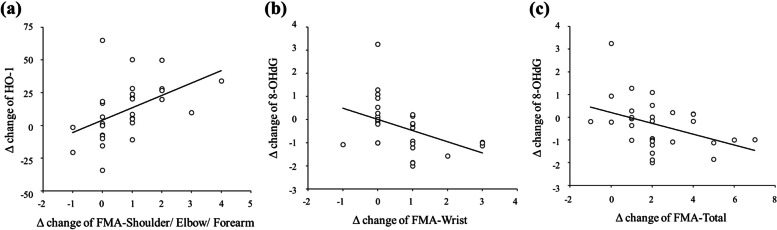
Relationship between changes of motor performance and serum biomarkers. **a** Correlation between the change of FMA-UE-Shoulder/Elbow/Forearm score and HO-1. **b** Correlation between the change of FMA-UE-Wrist and 8-OHdG. (C) Correlation between the change of FMA-UE-Total and 8-OHdG. FMA-UE: Fugl-Meyer Assessment for upper extremity

Baseline IL-6, ICAM-1, HO-1, and BDNF did not correlate with the improvement in any motor performance items. Interestingly, there was a significant positive correlation between baseline 8-OHdG levels and the improvement in FMA-UE-Total scores ( *r *= 0.463, *p* ≤ 0.01). Moreover, changes in HO-1 levels were positively correlated with improvements in FMA-UE-Shoulder/Elbow/Forearm scores (*r* = 0.478, *p* ≤ 0.01; Fig. 5a). Changes in 8-OHdG levels were related to improvements in FMA-UE-Wrist (*r* = − 0.436, *p* ≤ 0.05) and FMA-UE-Total (*r* = − 0.399, *p* ≤ 0.05) scores (Fig. 5b and c). No significant relationships were observed for other comparisons.

### Feasibility and safety

**Fig. 6:**
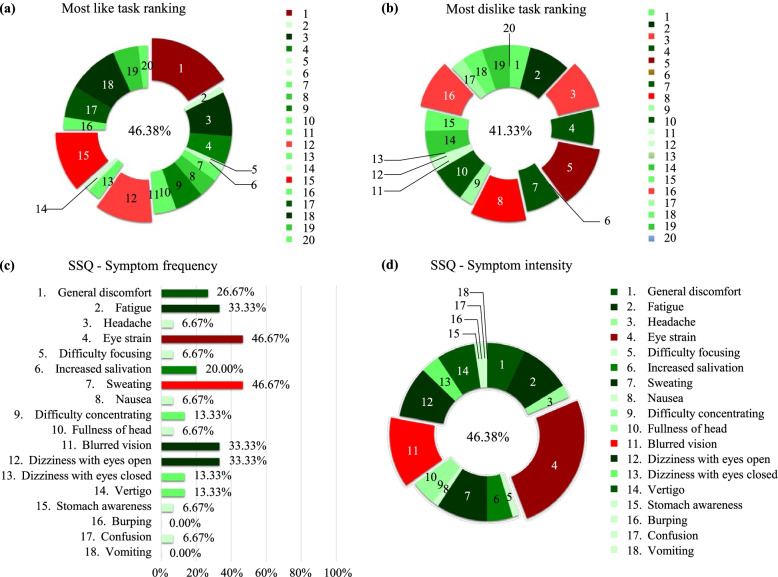
Results of the simulator sickness questionnaire (SSQ) and game-like rating. **a** Percentages of patients with uncomfortable symptoms. **b** Percentages of occurrence of SSQ symptoms. **c** Percentages of top favorite game rated by participants. **d** Percentages of top disliked game rated by participants

All participants completed the trial, and none experienced immersive VR before the intervention. Among the 20 activities, participants voted Scene 1 (19.57%) as the favorite activity, followed by Scene 15 (13.77%) and Scene 12 (13.04%), while Scene 5 (12.00%), Scene 8 (10.67%), Scene 3 (9.33%), and Scene 16 (9.33%) were less popular activities (Fig. 6a and b). The information of each scene is listed in the Additional file. As for satisfaction with the VRT, participants reported an average score of 4.5 out of 5 points. None of the participants reported a satisfaction score < 3.

In the VRT group, the total SSQ score ranged from 0 to 7, with a mean of 0.387. The most common adverse symptoms were eye strain (46.67%) and sweating (46.67%), approximately 26.6% of participants experienced both symptoms. Eye strain was the most intense (25.58%) followed by blurred vision (12.79%), fatigue (9.3%), sweating (9.3%), and dizziness with the eyes open (9.3%). Most symptoms were mild, except for eye strain and blurred vision, which were reported to cause moderate discomfort. Additionally, burping and vomiting were not reported (Fig. 6c and d).

The average RPE score for a single session was 12.4; personal RPE scores ranged from 11 to 13.5. Some participants reported that they felt as if they were exercising in the real-world during tasks in Scenes 1, 3, 4, 11, 15, and 20. Several scenes, such as Scenes 8, 9, 14, 16, and 17, were fast-paced and had virtual enemies, which made the sessions tense, and exciting. This training made some participants sweat and pant, but they did not report other uncomfortable symptoms.

## Discussion

This study aimed to investigate the effects of motor control training with a commercial, immersive VR system on upper limb motor function, inflammation, oxidative stress and neuroplasticity in patients with stroke. There were four main findings in our study. First, with respect to inflammation, a significant decrease in IL-6 levels was found after the intervention, despite the lack of significant group differences. Second, as for oxidative stress, significant differences between pre- and post-tests were found in both HO-1 and 8-OHdG levels, without significant differences between groups. Third, consistent with previous studies [11, 47], significant improvements in overall upper limb function and AROM were found after the intervention, while only elbow extension and forearm pronation were significantly different between groups. Forth, there were significant positive correlations between baseline 8-OHdG and the improvement in FMA-UE-Total scores, and between the change in HO-1 levels and the improvement in FMA-UE-Shoulder/Elbow/Forearm scores.

In our study, although VRT did not significantly affect BDNF expression level, a significant improvement was found after intervention in the VRT group. This result indicated that BDNF induction might be affected by a specific type of intervention or rehabilitation program. This is the first immersive VR-based study to report a significant increase in BDNF levels after motor control training; similarly, Koroleva et al. showed that BDNF concentration was significantly induced during augmented-reality-based motor training (average 45 days) [48]. On the other hand, our study reported the lack of significant relationships between BDNF level and motor function outcomes. One potential explanation may be that the increase may not be enough to reach significance in such short time period. In animal models, increasing BDNF expression by endogenous proteins and blocking BDNF expression by antisense oligonucleotide have shown positive and negative impact on motor recovery, respectively [49, 50]. However, the concentration of VRT-generated BDNF might not reach such a high level of endogenous protein (20 μg/g in rats), and it is unclear whether long-term continuous stimulation by VRT has beneficial effects on BDNF levels. Additional testing is required to monitor the changes in BDNF level over time rather than a few time points.

As we expected, IL-6 and 8-OHdG levels significantly decreased and the HO-1 level significantly increased after the intervention. Evidence has shown that the pro-inflammatory cytokine IL-6 increases after stroke, and higher IL-6 concentrations during the acute phase (< 7 days) after onset were associated with poor short-term and long-term outcomes in stroke patients [23–25]. Liu et al. found that serum IL-6 levels significantly decreased after evidence-based nursing interventions [26]. Therefore, the decrease in IL-6 may indicate that patients may return to a more homeostatic status after the intervention.

This is the first study to demonstrate significant changes in HO-1 and 8-OHdG levels after **an** immersive VR-based intervention of motor control training, as well as the significant relationship between these two oxidative stress biomarkers and functional outcome in patients with chronic stroke. Since redox homeostasis in the chronic stroke stage is relatively more stable than in the acute phase, our findings suggest that HO-1 and 8-OHdG may have a predictive value for motor function, and the reduced reactive oxygen species might be associated with HO-1 dependent pathways. Further work will be necessary to elucidate the role of HO-1 in patients with chronic stroke.

Consistent with previous studies, the present results showed that most participants in both groups had improved upper limb motor function after the intervention [10, 13, 14]. There were significant interactions in the subscale score of proximal upper extremity of the FMA-UE, total scores of the FMA-UE, and AROM of shoulder flexion. As shown in Fig. 2, these results indicated that the changes in these variables might be larger in the VRT group than in the COT group. Moreover, the AROM of elbow extension and the AROM of forearm flexion both showed intervention and time effects. These results also indicated that the VRT group had larger improvements than the COT group in the two AROM variables. In summary, our results in relation to upper motor function support the use of a commercial immersive VR system for motor control training if participants follow the researchers’ instructions.

We further compared the popularity of the 20 commercial VR activities. Scenes 3, 8, and 16 were three activities that participants’ least liked. There are two possible reasons. First, upper limb motor functions needed in these three activities were asymmetrical bilateral coordination, which may be more difficult than other activities only needing motor functions of symmetrical or reciprocal bilateral coordination. Second, it is counterintuitive to perform these three activities because different methods are used to perform the activities in the VR environment and in the real world. For example, in the three activities, participants picked up an object by pressing the trigger on the controller, and released it by holding the controller still.

In this study, age showed significantly negative association with the improvement of the hand score of FMA-UE. The result was supported by previous research reporting that age was a crucial predictor of good functional outcome in stroke patients [51]. In a large multicenter study, Knoflach et al. found better outcomes in stroke patients aged < 55 years [52]. Potential explanations are the regenerative capacity in the brain and angiogenesis [53]. Regarding other demographic and clinical characteristics, cognitive functioning, stroke subtypes, affected side, and years of education showed no significant relationship with motor performance and indicators of inflammation, oxidative stress and neuroplasticity. However, the results might be limited by the sample size. Further studies including a representative sample to identify responders to immersive VRT among patients with stroke are warranted.

Moreover, a commercialized immersive VR system has several advantages for clinical practice, such as being user-friendly and high accessibility, and for containing a wide range of well-developed games. Most importantly, the highly interactive entertainment nature of the VR can be used to enrich the training content and help boost the rehabilitation motivation of patients with stroke. Such advantages allow practitioners to seek and integrate their knowledge to meet the diverse rehabilitation needs of patients with stroke. For example, occupational therapists could integrate the principles of occupational therapy into VR activities, and thus achieve the patients’ rehabilitation goals.

In addition to functional and biological changes after receiving the training, our study also addressed the negative effects of VRT on participants. Several safety issues were identified based on the participants’ feedback and researchers’ observation. Some participants reported moderate eye discomfort (e.g., eye strain and blurred vision) and sweating after training. Previous studies using the VR modality in motor recovery training also reported similar side effects [14, 54, 55]. Therefore, breaks are needed during each training section, and the time for each training section may need to be limited to 60 min. Although side effects were reported in some participants after receiving VRT, generally, participants described high satisfaction with VR activities (an average score of 4.5 out of 5 points). Accordingly, as long as there is awareness of its possible side effects, VRT can potentially facilitate upper limb motor functions in patients with stroke.

Moreover, two observations from the researchers about the safety issues should be addressed during the training process, although there were no accidents due to falling or bumps. First, in the pilot trial phase, when participants moved to an undetectable space of the VR system, the VR scene would be paused or shaken, resulting in a feeling of sudden dizziness. Therefore, in formal trials, researchers should remind participants to remain within the detectable space for the VR system. When participants accidentally moved out of the space, we asked them to stop the movement and close their eyes until we helped them go back to the right space. Second, participants tended to lie on the devices presenting in the VR environment (e.g., a table or a shooting machine). This may increase the falling risk of participants. Therefore, it is better to place some devices such as tables or stable shelves in the corresponding places in the real environment.

Four limitations should be noted in our study. First, participants were a sample of patients with chronic stroke. Whether similar differences in serum biomarker levels are observed among patients with stroke and healthy adults remains to be examined. Second, inflammation, oxidative stress, and spontaneous motor recovery are relatively high and unstable during the acute phase; these may drop, or be maintained at low-levels for months or even years. To identify a biomarker sensitive to VRT at the chronic stage, further research is needed to examine the extended time course of VRT by assessing more stroke biomarkers at regular intervals across a longer period. Third, five detected molecule biomarkers showed no significant differences within groups, although the BDNF change trends were the opposite in the two groups. These data must be regarded as preliminary because of the relatively small sample size, and the lack of BDNF polymorphism information. Future longitudinal studies with a larger sample size are required to confirm the present findings identify suitable subjects for the VRT. Fourth, several variables related to stroke data were not collected in our study (e.g., use of tissue plasminogen activator at the onset of stroke) and the sample size was small; thus, we may not have been able to identify more responders to the VRT.

## Conclusion

To the best of our knowledge, this is the first study to examine the effects of an immersive VR-based intervention of motor control training on pathophysiological molecules in chronic stroke patients. Our results suggest that HO-1, 8-OHdG, and BDNF might be potential serum biomarkers for VR-based interventions in chronic stroke patients. This study also indicated that general commercial VR applications may provide therapists with more diversified options for post-stroke rehabilitation, as they are effective and safe under the guidance of occupational therapists. The clinical recommendations of the study are the application of immersive VR-based training on upper extremity motor performance and the detection of serum biomarkers of oxidative stress and neuroplasticity as potential outcome measurement after motor control training in chronic stroke patients.

## Supplementary Information

**Additional file 1.** Information of selected commercial game.

**Additional file 2.** Scenarios of selected commercial games.

**Additional file 3.** Trial protocol.

## Data Availability

Raw data were deposited in Mendeley Data [Effects of Virtual Reality-based Motor Control Training on Inflammation, Oxidative Stress and Neuroplasticity in Patients with Chronic Stroke: A randomized controlled trial]. This dataset can be accessed from https://data.mendeley.com/datasets/cbd8bnhg3n/1

## Abbreviations

VR: Virtual reality
COT: Conventional occupational therapy
VRT: Virtual reality training
AROM: Active range of motion
HMD: Head-mounted display
FMA: Fugl-Meyer Assessment
UE: Upper extremity
IL-6: Interleukin 6
ICAM-1: Intracellular adhesion molecule 1
HO-1: Heme oxygenase 1
8-OHdG: 8-hydroxy-2-deoxyguanosine
BDNF: Brain-derived neurotrophic factor
SSQ: Simulator Sickness Questionnaire
RPE: Rating of Perceived Exertion
