# Supplementary material for: Effects of virtual reality-based motor control training on inflammation, oxidative stress, neuroplasticity and upper limb motor function in patients with chronic stroke: a randomized controlled trial
Source: BMC Neurol. 2022 Jan 11;22:21. doi: 10.1186/s12883-021-02547-4 (PMC8751278; doi:10.1186/s12883-021-02547-4)
Supplement: Supplementary file 1 — Additional file 1. Information of selected commercial game. [file 12883_2021_2547_MOESM1_ESM.docx]

**Additional file 1. Information of selected commercial game**

| Scene-serial | Game rule based on the original game. | Therapist assigned tasks (using the virtual objects appearing in the virtual scene) |
| --- | --- | --- |
| 1 | Batting the football with racket. | To complete specific level.  To practice with specific hand or both hands. |
| 2 | Splatter targets with squirt guns, let the balloon burst | To complete a specific amount of balloon.  To practice with specific hand or both hands. |
| 3 | Set the board with number ablaze by shooting the flaming arrows. | To achieve specific score.  To aim at particular targets (fixed or moved target, or specific number). |
| 4 | Break the balloon with sword. | To hit a specific color of balloon.  To try to get highest score. |
| 5 | Shooting basket in front of the basketball machine. | To throw the ball to specific target position.  To try to get the highest score. |
| 6 | Throwing the gift of different colors to the horn of corresponding color. | To find specific object in the game scene, for example: toys, books, balls….  To throw the gifts with specific color to the target horn. |
| 7 | Pressing the button on a virtual table to make the virtual octopus fall from the sky.  Throwing the virtual octopus to the wall with ring target. | To hit specific ring or color on the wall. |
| 8 | Pressing the button to create virtual snowball.  Throwing the snowball on the snowman who appears from time to time.  Raising hand to block the snowball thrown by the snowman. | To practice a specific action described in game rule.  To act against the attack from the enemy, and try to survive longer in the survival mode.  To try to get higher score by hit snowman. |
| 9 | Catching the virtual car or human and eat or throw or smash it. | To practice specific action described in game rule. |
| 10 | Smacking the moles that light up with virtual mallets. | To practice with specific hand. |
| 11 | Punch the target moving towards the player. | To try to get the highest score. |
| 12 | Hit the clock hanging on the ceiling. | To practice with specific hand. |
| 13 | Shooting the fixed targets (dishes or vase). | To aim at specific targets. |
| 14 | Shooting the moving targets (dishes or vase). | To try to get the highest score. |
| 15 | Shooting different enemy with corresponding gun. | To shoot a specific type of enemy. |
| 16 | Shooting or beating the ghosts with gun. | To practice specific actions described in game rule. |
| 17 | Shooting the enemies and dodging to the side when they attacks. | To try to get the highest score by shooting the enemies.  To dodge attacks to survive longer.  To practice with specific hand. |
| 18 | Blending the beverages ordered from virtual customer. | To try to get the highest scores. |
| 19 | Observing the virtual environment without specific requirement. | To take specific beverages and observe them.  To make customized drink by several specific beverages with specific ratio.  To drop coins into the slot machine and pull the handle. |
| 20 | Slicing the fruit that spurted from the cracks on the ground. | To experience at least two modes, with and without bomb.  To slice specific fruit. |
| The above VR applications can be found in steam or VIVE platform. | | |
