# Supplementary figures and images for: Effects of virtual reality-based motor control training on inflammation, oxidative stress, neuroplasticity and upper limb motor function in patients with chronic stroke: a randomized controlled trial
Source: BMC Neurol. 2022 Jan 11;22:21. doi: 10.1186/s12883-021-02547-4 (PMC8751278; doi:10.1186/s12883-021-02547-4)

**Additional file 2. Scenarios of selected commercial games.**


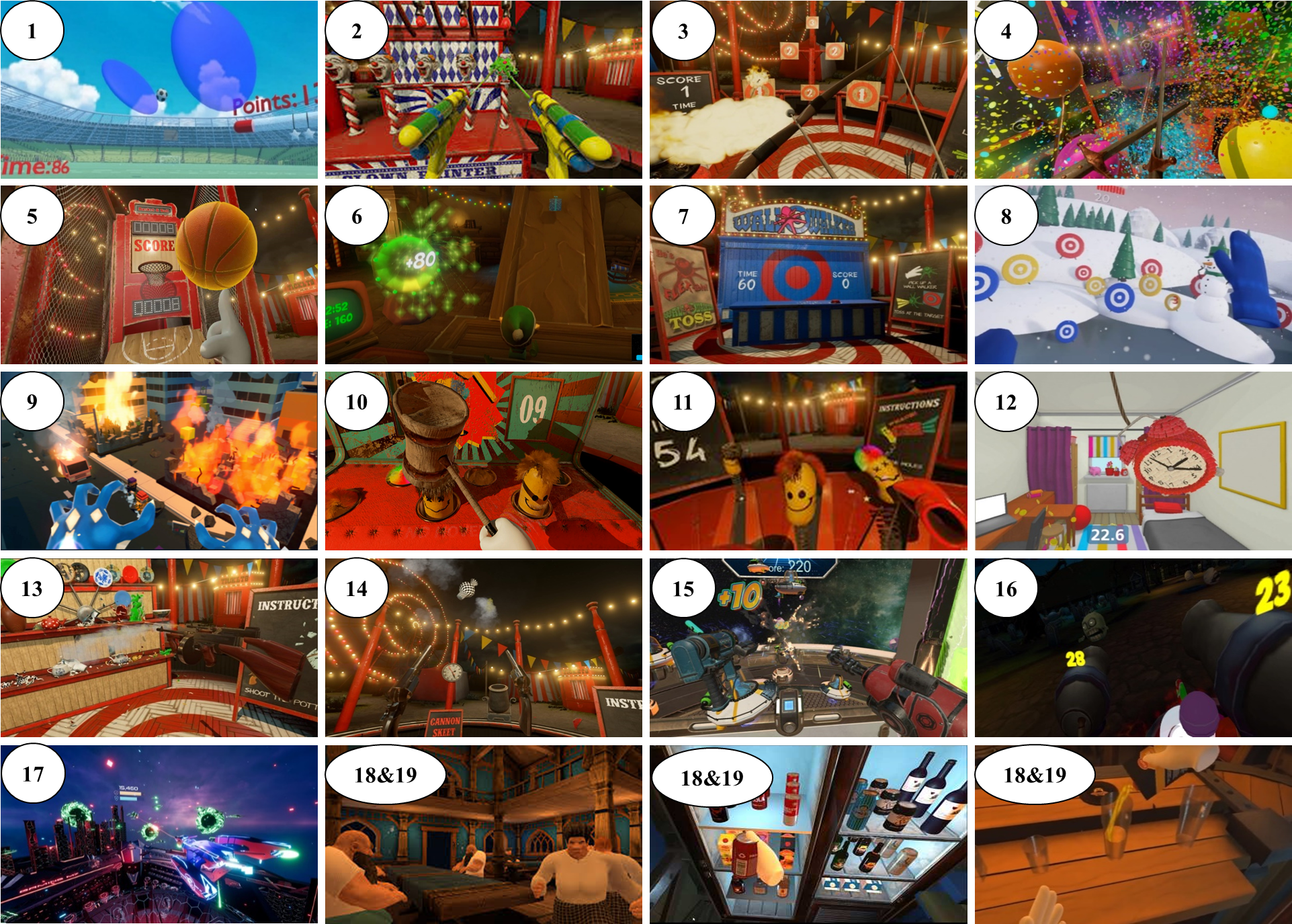

Supplement: Supplementary file 2 — Additional file 2. Scenarios of selected commercial games. [file 12883_2021_2547_MOESM2_ESM.docx]
