# Supplementary material for: Effects of virtual reality-based motor control training on inflammation, oxidative stress, neuroplasticity and upper limb motor function in patients with chronic stroke: a randomized controlled trial
Source: BMC Neurol. 2022 Jan 11;22:21. doi: 10.1186/s12883-021-02547-4 (PMC8751278; doi:10.1186/s12883-021-02547-4)
Supplement: Supplementary file 3 — Additional file 3. Trial protocol. [file 12883_2021_2547_MOESM3_ESM.docx]

**Additional file 3. Information of the trial protocol**

Full details of the trial protocol of this study is available at https://protocolexchange.researchsquare.com/
